# On the Advance of the Asiatic Cholera; with Suggestions for Its Treatment

**Published:** 1848-03

**Authors:** Charles W. Bell

**Affiliations:** Manchester


### SELECTIONS

FROM

### AMERICAN AND FOREIGN JOURNALS.

---

*On the advance of the Asiatic Cholera; with suggestions for it Treatment.* By CHARLES W. BELL, Esq., Manchester.—The near approach of the cholera, together with your challenge to the profession, to determine upon the principle which is to guide the treatment of that disease, induce me to venture on expressing the views which peculiar circumstances, and much experience of its management, have led me to form; and to state the practice which, all throughout the progreas of the cholera that appeared in Persia in 1842 and 1843, proved successful in some thousand cases.

It is true that the type of disease in that epidemic was mild in the generality of cases, but it was the commencement of the identical cholera that is now approaching us; and, if changed now, is changed probably only in degree, not essentially in its nature; and as treated by the native practitioners was almost always fatal.

It reappeared irregularly in Persia in 1844–45, and again, with greater virulence, in 1846; while in the present year, forsaking the Eastern provinces of that country which it had scourged, it passed on, as in 1831, to the Russian provinces of Georgia and Circassia, and into the Turkish province of Armenia.

In Southern Asia, after confining its attacks for two years to the Southeastern provinces of Arabia Mesopotamia, it has now advanced and crossed the Red Sea into Egypt on the one hand, and on the other overspreading Asia-Minor up to the coast of the Levant; now threatening Greece, and it is said has already appeared in Constantinople. Meanwhile passing to the north of the Caucasian range, it has arrived at Riga, and also penetrating Circassia from Teflis, it has crossed the mountains, and arrived at Varouish, the most central town of Southern Russia.

Again passing through Turkish Armenia, it reached Trebisonde, and thence crossing the Black Sea, arrived at Varna, the mouths of the Danube and Odessa; and already single cases are reported at Vienna.

Thus instead of the limited course which it pursued in 1831–32, confined to Russia, Prussia and Denmark, till it finally reached England, and only returning in 1843–44, and 45 upon Austria, Saxony, Southern Germany, France and Italy, and attacking them in the irregular manner in which it is wont to revert to places that it had left intact in its first progress,—it is now advancing upon the whole of Europe at once by five different routes, and appeared in as many points in the extended line between Greece and Riga.

We have, therefore, reason to fear that its future progress may be marked by a virulence proportioned to its extended front and uniform progress. Under such circumstances, I hold it to be an imperative duty in any one who has had an opportunity of studying the disease, to add what he can to the imperfect knowledge that we possess on the subject: and I venture to hope that these considerations will excuse me for throwing aside much of the diffidence that I should otherwise have felt in publishing my own views and until they should be further confirmed.

It was my good fortune first to study the disease under my cousin, Mr. G. H. Bell, in Edinburgh.—author of what appears to me to be incomparably the best work on the subject that I have met with. I afterwards saw the disease in London, when it was the fashion to treat it on Dr. Stevens' system of endeavoring to supply the exuded serum of the blood by the administration of salt and soda. After this, all the experience I had of it was a chance case in Bombay, till 1842, when a series of epidemics of peculiar character commenced in Persia, where I was attached to the embassy, and was in medical charge of the Persian army.

The first of these was dysentery; then a peculiar periodical disease, till then undescribed, attended with intense disturbance of the circulation and nervous system: which also appears to have been observed at Strasburg. Then came tropical remittent, then congestive ague: and this finally terminated in true cholera.

To this series of disease I am indebted for being led—as I

cannot but think I have been—by a species of natural analyses, and by easy transitions, to a clearer insight into the nature of the disease; and to a practice, unbiassed by theory, and simply arising out of the process of accommodating my remedies to the changing type of disease: which then proved successful in every case I attended, where the feet had not already become warm, while the legs and body remained cold.

This is a symptom which my whole experience teaches me to consider as a sign of actual death. In this most authors of experience bear me out, and I have invariably found that every interference with the patient who presented this fatal symptom only increased the spasms and suffering, and hastened the consummation.

Almost every intelligent author on this subject has classed cholera with the cold fit of ague—as, indeed, the whole of its features render nearly inevitable; and viewing it in this light, the rationale and use of bleeding, as recommended by Mr. Bell and other authors, could not be better laid down than it is in his work on Cholera Asphyxia. Yet among these authors I have searched in vain for a single statement that cholera is—what I am satisfied it will invariably be found to be—a congestive ague of quotidian type. All whose works I have read consider it as, *in its whole course*, merely the cold fit of an ague, and that the fever which occasionally succeeds to it on recovery is the hot stage.

In my belief, no single paroxysm of ague of any kind ever occupied more than twenty-four hours in passing through all its stages, and, according to my experience, (whenever its progress is sufficiently slow to run that course) invariably completes an entire paroxysm within that period, consisting of the congestive or cold stage, and the remission. The stage of reaction which follows congestion can scarcely be said to exist in cholera, and the sweating stage, or stage of relaxation, is only occasionally perceptible, being so little marked as to be nearly undistinguishable from the short period of remission or intermission, but in every case of the epidemic which I had most opportunity of observing, there was invariably a diurnal remission and quotidian accession; and I am greatly inclined to believe this universal in all forms of cholera, where not prevented by previous death or cure.

This, sir, will doubtless appear to you a very bold assertion, and I am aware that it is one which, without the stethoscope at the bed side of the patient, will be most difficult to verify, seeing that the chillness of the surface is almost as great in this stage as in that which precedes it: at the same time it is very valuable, and will at once indicate a principle for the guidance of the practitioner, which will induce the utmost confidence in his plan of treatment.

The symptoms of these stages are so little marked as to require the most attentive observation to distinguish them, and until the opportunity shall present itself of actually looking for them, I do not expect to be credited, and all that I can hope for at present, from those most conversant with the disease, and who have not as yet entertained this view, is, that they will at least give it their consideration, and try to recollect whether in the more protracted cases that have fallen under their treatment, they have not observed that the patient, after tossing about for many hours in an agony of suffocation, seems at last exhausted, and after long jactitation and continuing to throw off every covering he at last for a short period remains quiet and submits to the load of the bed-clothes. This temporary repose is in most cases almost the only symptom, except a more tranquil action of the heart, that marks the intermission, and forms the most obvious and almost the only guide to the essential part of the treatment of a severe case. In a very short time this period of comparative tranquility ceases—the patient begins again to yawn, to throw up his arms like one bleeding to death, and in a few minutes more to toss about again, and show every symptom of suffocating agony.

These symptoms of the return of the congestive stage have, in the mass of my cases, appeared exactly twenty-four hours after the first accession, insomuch that I invariably made a point of seeing my patient some time before the hour of the commencement of the first attack, and selected it as the moment for the successful use of the lancet, where that fearful but powerful remedy was necessary. But does it indeed require all the sudden and perfect restoration to health which attends its use, to compensate for the first anxious moments of suspense until the blood begins to trickle, or to reconcile the practitioner to such a measure? for when the first few drops ooze from the wound, they seem the harbinger of immediate dissolution; but when its flow is once established, and the patient is at once restored to health, no triumph of art and just principle can exceed it. Latterly, however, I was not in the habit of waiting so long; when the repose of the patient had been well marked for a short time, I bled, with much less difficulty in obtaining blood—with much less struggle and danger to the patient, and with equal success—forestalling, instead of combatting, the anticipated period of fatal congestion.

In the remittent, and afterwards in the intermittent fevers which preceded the appearance of cholera in 1842, I found quinine alone, so far from relieving the symptoms, invariably aggravate all of them. I was led by circumstances to administer it in combination with iron, with complete success; and when by almost insensible transitions the epidemic merged into cholera, I continued the same practice. I had the pleasure to find that the first doses of a combination of sulphate of quinine, sulphate of iron, a few drops of sulphuric acid, and often a little sulphate of magnesia dissolved in water, immediately checked the vomiting and diarrhœa; and this I have found equally successful in the only case of true cholera I have met with in England. It occurred in the autumn of last year. The woman, who had previously suffered from the epidemic of 1833, was attacked on her way from Crewe to Manchester, and had been laboring under the cholera for eight hours. I found her cold, blue, purging and vomiting the rice-water evacuations, and cramped, with cold clean tongue, and feeble pulse. She had taken large doses of morphia, which were immediately rejected; but the first dose of the above medicine checked the vomiting, and she was well in a few hours.

In all the mild cases in Persia this remedy was of itself sufficient to cure: it was repeated very frequently in general, but some of the most rapid cures I afterwards learned had been effected by swallowing at once the whole of what I had intended for nine doses. Its good effects were generally immediately indicated by diminished anxiety, returning warmth, proceeding from the chest to the extremities, recommencing secretion of urine; but in cases of severer character it was found necessary to relieve the congestion by the abstraction of blood from a vein; and in doing this all depends upon the period at which bleeding is resorted to. If early in the congestive stage, or just previous to its second accession, it is invariably successful; if just as the congestive stage is passing off, when the pulse begins to acquire a little power, it is invariably fatal.

Before proceeding further, or considering the appropriateness or the contrary of the remedies most lauded in cholera, viz: opium, stimulants, antimonials, artificial heat, or cold affusion,—it will be necessary to consider what takes place in an attack of cholera, that, from its etiology, a theory of treatment may be deduced to point the indications and to defend the treatment which I have ventured to advise; for though arrived at rather by practice than by theory, it is impossible that it can command attention unless borne out by reason.

1st. With many able authors, I hold cholera to be merely a form of ague; and, in addition, I maintain its type to be quotidian.

To determine the nature of ague, therefore, if this position be true, is to determine both the nature and treatment of cholera. To do this it is necessary to have a clear conception of what is really essential in an ague.

In the first place, I may venture to premise that I have enjoyed very ample opportunity of seeing ague in every variety of type and degree of severity; yet I have never seen a single attack of any kind of ague in which the whole cycle of its stages—that is, in which each individual paroxysm—was not completed within twenty-four hours. I have often witnessed several paroxisms completed within that time, but never one incomplete; and I have never met with a description of an ague in which the paroxysm exceeded twenty-four hours duration, except where the author would appear to have considered Asiatic cholera to be such an ague.

2dly. The congestive or cold stage is the only essential one which is never wanting in ague. It may be severe or slight, partial or general,—be with or without shivering;* but in ague, and every disease allied to it, such as masked and misplaced agues, as they are called, periodical headache, and neuralgia and all the remittent fevers I have yet met with, this stage is never wanting, though sometimes obscure. The second or hot stage may or may not occur; reaction may, and often does, take place quietly, and without fever, and unsucceded by a period of relaxation or sweating. This condition frequently occurs in what is called the fortnight ague—a form with which all who served in the late campaigns in Scinde are well acquainted, where the shivering is severe and hysterical, but where the febrile and sweating stages are often entirely wanting; and yet this is the form most remarkable in its periodicity, and in its return, for one or more days, every fortnight, just previous to the lunar changes.

---

* I have latterly been in the habit of considering shivering as a favorable symptom—an indication that the power of the heart is sufficient to overcome the congestion. If this be insufficient to produce shivering, the after symptoms are severe; if such as to overpower this symptom, the congestion itself is always dangerous.

In other forms, more especially the quartan, all three stages generally prove exceedingly severe, and in this form the period of relaxation my perhaps pass the twenty-fourth hour from the accession, but I have not seen it do so, and I should be inclined to define ague as a paroxism of congestion of the internal veins, subsiding wtihin twenty-fours hours, and often followed by febrile action and relaxation of the capillaries. It appears to consist in a gradual change in the action of the extreme capillaries, and apparent constriction of them, by which the blood is rapidly driven inward upon the great veins. When this has reached to such a point as to oppress the action of the heart, yawning first, and then shivering, or a sense of suffocation and pain in the præcordia, are the indications of oppressed circulation, and of the commencing effort of the heart to overcome the mass of blood which is stifling it. If by application of tourniquets to the limbs, or by bleeding, part of the blood which is rushing from the extremities to increase this congestion is prevented from reaching the great veins,—the heart, excited to increased action, is enabled by this relief more quickly to overcome the obstruction and restore the balance of the circulation, and the paroxism passes off. If not thus mechanically aided, the heart, after a severe struggle to maintain the circulation during the period of constriction, is at length relieved, by this nervous disturbance or spasm of the capillary circulation passing off of itself, and then the heart and arteries, so long excited by the struggle, maintain for a time their increased action after the obstruction in the capillaries is removed, and produce apparent febrile action. Presently this excitement subsides, the vessels become relaxed, and sweat succeeds. The vessels continue in this state for a longer or a shorter period, according to circumstances, till they at length recover their ordinary tone and action in the intermission. This fever, however, is not fever properly so called, but reaction; and the sweating not critical or essential, but relaxation. The cold stage is alone essential, and is the physiological cause of the subsequent stages.

If this be admitted, it is obvious that our whole attention must be devoted to the study of the phenomena of aguish congestion, in its commencement, its progress, its mechanical effects on the organism, and the natural means by which it subsides or is overcome; and, lastly, in its tendency to periodical return, if we would obtain a clear perception of the nature of cholera. Before, however, we enter into these considerations, and the ratio medendi, it may be permitted to us to diverge for a moment to consider the question of contagion, and inquire how far ague and cholera resemble or differ from the whole class of contagious fevers, so as to justify us in chiming in with the popular opinion of its infectious nature, or of entertaining views to the contrary; for where popular feeling is stong on such a subject, abundant evidence is easily found to support their opinion of apparent facts.

Of the exciting cause of cholera we know little or nothing, and not a great deal of ague. The latter is known as a disease of marshy countries, but it is also the most prevalent disease in the driest countries in the world; for example, the whole of central Asia, the deserts of Arabia and of Africa, of some of the most elevated regions of Italy, etc. etc. A hundred years ago ague prevailed throughout Britain and Ireland, but it has now equally ceased in the most improved and well drained parts of Britain, and the most neglected of Ireland; and no one can say that an improved mode of living among the peasantry of that country is the cause of its disappearance; yet no one considered ague infectious. Marsh miasm, then, if a cause, is evidently not the only cause of ague; and among others, one of the most obvious, in the eastern countries at least, is subterranean volcanic action; whether by means of exhalations, or the electrical effects of subterranean chemical action, it would be difficult to say, but I have seen it and remittent too often precedes or arise with earthquakes, to have any doubt on this subject. Whatever its cause, however, most people are inclined to attribute it to a terrestrial origin; but, however we view it, it must be conceded that it was once endemic in England, and is not so now; yet ague will march across countries as steadily and extensively as cholera. The remittent fever of 1842, in Scinde, ending in ague, proceeded in a direct line through Beloochestan, right across Persia, Georgia, Circassia, and southern Russia, and gradually losing more and more of its intermitting character, appeared in Edinburg as a modified remittent, with a tendency to periodical relapse, and is described in your journal under the title of the Scotch Epidemic. This remittent tendency cotinued in 1844 and 1845 to influence the type of fever in Scotland, Manchester and Liverpool in these years.

In 1843, in the Asiatic part of its course, this remittent and intermittent assumed the shape of cholera. In 1846–47, cholera there became a scourge, while in this country we only slightly perceived the influence in the increased tendency to intermitting neuralgia, and the secretion of oxalates in the urine, etc. If, then, cholera is propagated by contagion, is it possible that its approach alone, and before its advent, should so markedly be perceptible in the character of the prevalent diseases, or could it continue to influence them after its disappearance, when confessedly no contagion prevails? Yet it has been remarked by some of our most experienced authors that, ever since the first approach of cholera, the fevers in this country have materially changed in their type, and the use of the lancet in fever, formerly so beneficial, has now become obsolete. These considerations tend to show that (ague and cholera being so nearly allied) there is no reason why cholera should not for a cycle become as endemic in this country as ague formely was, and as it has in fact, become in India since its appearance there in 1767.

But let us examine the action of the poison, whatever it may be that produces ague or cholera; in its effects on the system, as compared with those of contagious fevers, including the exanthemata, and other fevers produced by animal poisons, and whether there is any evidence of its elimination from the system by similar febrile action.

I presume I shall not be going too far in considering continued fevers as the reparative process by which nature eliminates the poison by appropriate organs—the skin, the bowels, etc.; and it will not invalidate this position, that death occasionally occurs through the violence with which this action falls on a particular organ; for if the organs fail to eliminate the poison, and thus protect the nervous centres, death takes place through the nervous system.

It is not so in ague and cholera; in the latter the brain is the last to die. There is no evidence of any poison being eliminated by the action, or any special secreting organ being affected. An ague fit will sometimes pass off without fever or sweating, a cholera attack sometimes without even purging, and leave no evidence of a poison lurking in the system. Whatever the cause of the disturbance of the circulation that produces the congestive stage in these diseases, the fact of an immediate cessation, or a complete intermission of its effects, is almost conclusive that it cannot be, as in other fevers, a poison circulating in the blood, exciting a reparative action, but the effect of a cause acting on the powers which circulate the blood, independently of a change in the blood itself, and most dissimilar to anything that we perceive in the ordinary course of contagious fevers.

We can scarcely doubt that the congestion of ague takes place through an influence of the sympathetic nerve on the minute capillaries; but till we are better acquainted with the powers which circulate the blood, with the changes which that fluid undergoes in passing through them, and with the effects of electricity upon them, we cannot expect to arrive at a knowledge of the exciting cause of these diseases, but we do not, in the nature of the disease, find a single argument for believing that either ague or cholera eliminated from the system any morbid poison to render them communicable from man to man: although in both we find some reason to believe that that powerful agent, electricity, of which we are as yet so ignorant, has much to do with them, and perceive some evidence of a connection with the terrestrial currents as affecting their periodicity, and with the electrical phenomena that accompany the lunar changes, etc. as effecting their relapse.

The direct evidence in favor of non-contagion has always appeared to me infinitely to preponderate over that to the contrary; and know none more conclusive than the fact of its penetrating the triple cordon sanitaire established by the Prussian Government on the Oder in 1831 at exactly the same rate, four German miles a day, that it proceeded at both before and after its encountering what was expected to prove so formidable a barrier to its progress.

While on this point, however, it would be wrong to pass over a remarkable point which many cases published in the periodical medical journals confirm—that where fever is sporadically induced by accumulated filth, especially decaying vegetable matter, such as stable manure, the type of fever that results is the most exaggerated specimen of the type of the fever that prevails at the time; and thus it happens that people from the same neighborhood are so rapidly affected by a prevailing non-contagious malady as to present every appearance of having received it by contagion.—*London Medical Gazette.*

